# Xtreme Everest 2: unlocking the secrets of the Sherpa phenotype?

**DOI:** 10.1186/2046-7648-2-30

**Published:** 2013-10-23

**Authors:** Daniel S Martin, Edward Gilbert-Kawai, Denny ZH Levett, Kay Mitchell, Rajendra Kumar BC, Michael G Mythen, Michael PW Grocott

**Affiliations:** 1 UCL Centre for Altitude, Space and Extreme Environment Medicine, Portex Unit, Institute of Child Health, 30 Guilford Street, London WC1N 1EH, UK; 2 Division of Surgery and Interventional Science, University College London, 9th Floor, Royal Free Hospital, Pond Street, London NW3 2QG, UK; 3 NIHR University College London Hospitals Biomedical Research Centre Research & Development, Maple House 1st floor 149 Tottenham Court Road, London W1T 7NF, UK; 4 Integrative Physiology and Critical Illness Group, Clinical and Experimental Sciences, Mailpoint 810, Sir Henry Wellcome Laboratories, Faculty of Medicine, University of Southampton, University Hospital Southampton NHS Foundation Trust, Tremona Road, Southampton SO16 6YD, UK; 5 Anaesthesia and Critical Care Research Unit, University Hospital Southampton NHS Foundation Trust, Mailpoint 27, D Level, Centre Block, Tremona Road, Southampton SO16 6YD, UK; 6 NIHR Southampton Respiratory Biomedical Research Unit, Southampton SO16 5ST, UK; 7 Nepal Health Research Council, Ramshah Path, Kathmandu 44601, Nepal

**Keywords:** Altitude, Oxygen, Hypoxia, Sherpa, Critical care, Intensive care, Microcirculation, Mitochondrion, Nitric Oxide, Epigenetics

## Abstract

Xtreme Everest 2 (XE2) was part of an ongoing programme of field, laboratory and clinical research focused on human responses to hypoxaemia that was conducted by the Caudwell Xtreme Everest Hypoxia Research Consortium. The aim of XE2 was to characterise acclimatisation to environmental hypoxia during a standardised ascent to high altitude in order to identify biomarkers of adaptation and maladaptation. Ultimately, this may lead to novel diagnostic and treatment strategies for the pathophysiological hypoxaemia and cellular hypoxia observed in critically ill patients. XE2 was unique in comparing participants drawn from two distinct populations: native ancestral high-altitude dwellers (Sherpas) and native lowlanders. Experiments to study the microcirculation, mitochondrial function and the effect that nitric oxide metabolism may exert upon them were focal to the scientific profile. In addition, the genetic and epigenetic (methylation and histone modification) basis of observed differences in phenotype was explored. The biological samples and phenotypic metadata already collected during XE2 will be analysed as an independent study. Data generated will also contribute to (and be compared with) the bioresource obtained from our previous observational high-altitude study, Caudwell Xtreme Everest (2007).

## Background

The study of human adaptation to high altitude has a long history [1]. We have proposed that data obtained from individuals at high altitude may increase our understanding of the pathophysiological effects of hypoxaemia in patients at sea level [2]. Tissue hypoxia secondary to hypoxaemia affects many patients admitted to intensive care units and may contribute to organ failure and eventually death. Our understanding of how tissues adapt to a lack of oxygen during disease is somewhat limited, and inter-individual variation in the response to hypoxaemia is marked. Due to the heterogeneous nature of critically ill patients, it is often difficult to elucidate a clear signal because of the high level of background pathophysiological noise; such that complex physiological studies in this group are limited in the conclusions they can draw. Being able to target specific mechanisms in the critically ill may be facilitated by preliminary studies in healthy volunteers ascending to altitude, in which a graded exposure to the environment hypoxia during ascent creates a paradigm for pathological hypoxaemia. Comparison of metabolic and physiological signals in ascending volunteers will yield information that other models, such as animal and cell line varieties, cannot generate. Systematic and comprehensive investigation of human adaptation to high altitude therefore provides a robust approach to driving clinical research in critically ill patients.

In 2007, the University College London Centre for Altitude Space and Extreme Environment (CASE) Medicine conducted the largest comprehensive prospective observational study to be performed at altitude: Caudwell Xtreme Everest (CXE) [3-5]. This study aimed to explore inter-individual differences in the response to hypobaric hypoxia. In particular, we hypothesized that (1) mechanisms distinct from those related to global oxygen transport play an important role in determining performance at high altitude (e.g. metabolic efficiency and microcirculatory blood flow) and that (2) genotype differences would explain a substantial proportion of intra-individual variation in environmentally induced phenotypes (gene-environment interactions). Results from CXE included novel findings in relation to microcirculatory blood flow [6,7], mitochondrial biology [8-10] and plasma nitrogen oxides [11]. These data led in turn to the core hypotheses for Xtreme Everest 2 (XE2). In XE2, the function of the microcirculation, mitochondria and nitric oxide metabolism were explored in two different population cohorts: lowland residents (primarily from the UK) and Sherpas. An important addition to our previous work has been the study of epigenetic profiles in all participants.

Data from CXE suggest that in lowlanders, there is significant inter-individual variability in changes in nitric oxide biology during ascent to high altitude [11]. Subgroup analysis of volunteers ascending to 5,300 m suggested higher erythropoietin, nitrate and cGMP levels at only 3,500 m in participants with considerable extreme altitude experience compared to those without [11]. Whilst this might simply be the result of self-selection, it may alternatively represent evidence of epigenetic imprinting from previous high altitude exposure. Epigenetic change, the ability to influence genetic transcription and translation through environmental stimuli, may provide a means of identifying candidate biochemical markers of successful adaptation to high altitude and thereby lead to viable pharmacological interventions that may benefit hypoxaemic patients. A pilot study of identical twins was therefore included as a supplementary component to the project in order to assess the methodological feasibility of future epigenetic research at high altitude.

### Xtreme Everest 2

It is possible that Sherpas have additional phenotypic advantages that are not evident even in high-performing lowland individuals at altitude. Thus, studying them in comparison to a cohort of lowland volunteers may identify traits that are difficult to tease out when looking only at lowland participants. As direct descendants of highland Tibetans who may have dwelt at an altitude of over 4,000 m for the past 20,000 years, the Sherpas of Nepal display exceptional phenotypic adaptation to this environment. The remote location of the Tibetan plateau also means that gene flow from other populations is likely to be minimal. Famed for scaling the Himalayan Giants whilst supporting lowland mountaineers, Sherpas demonstrate a high degree of physical resilience to hypoxia carrying heavy loads with apparently minimal effort. Whilst genetic inheritance and natural selection are likely to have led to traits that favour survival at high altitude, the physiological basis underlying their performance remains elusive. Of note, they have not been shown to demonstrate augmented systemic oxygen delivery, in contrast to what is commonly seen in lowland elite athletes at sea level. Strikingly, when compared to other resident high-altitude populations who have inhabited such heights for shorter periods of time (Andeans and Ethiopians), Tibetans demonstrate the lowest haemoglobin concentration [12,13]. The apparent lack of importance of systemic oxygen delivery in Tibetans/Sherpas suggests that factors in the peripheral compartment of the oxygen cascade may be of importance. Furthermore, even on descent to a lower altitude, Tibetans continue to demonstrate lower aerobic energy expenditure (greater economy of locomotion) during exercise than control participants [14]. Specifically, the microcirculatory-mitochondrial unit may be the site of beneficial adaptations in Sherpas. Preliminary data suggest that peripheral blood flow is markedly increased in highland Tibetans compared to lowland populations and that nitric oxide metabolism may play a role in this [15].

The XE2 study design shared a core dataset and ascent profile with CXE [5] but added more detailed characterisation of key pathways (microcirculatory blood flow, cellular respirometry, epigenetic change) and the comparison of different populations. Baseline measurements were obtained in London (50 m) for the lowlander participants and in Kathmandu, Nepal (1,300 m) for Sherpa participants. During March and April 2013, all participants ascended to the base camp of Mount Everest (5,300 m) in groups of up to 14, on an identical ascent profile to each other and, importantly, to the participants studied in CXE. This strategy ensured that the physiological challenge was matched for all participants and that differences detected between participants would be attributable to their individual phenotype, rather than variation in the magnitude or duration of exposure to hypobaric hypoxia. Data were obtained in dedicated laboratory facilities at 3,500 m (Namche Bazaar) and 5,300 m (Everest Base Camp) on ascent, and in the majority of participants at 1,300 m (Kathmandu) on descent. The conduct of XE2 would not have been possible without close collaboration with the Nepal Health Research Council and Nepali collaborators. They supported us in the preparation of ethical and research governance documentation, the recruitment and consenting of Sherpas, and subsequently helped with translation in the laboratories.

Detailed phenotypic characterisation included measurements of microcirculatory blood flow and tissue oxygenation by forearm plethysmography, near-infrared spectroscopy, tissue laser Doppler and side-stream darkfield imaging of the sublingual microcirculation. Mitochondrial function was studied with real-time cellular respiration measurements in fresh skeletal muscle samples, as well as metabolomic, proteomic and lipidomic screening of plasma, skeletal muscle and urine samples. Nitrogen oxide metabolism was studied in multiple compartments including plasma, urine, exhaled breath and saliva. These data will be combined with a core physiological dataset including daily physiological dairy incorporating simple cardiorespiratory variables and symptom scores (e.g. Lake Louise Score) [16] and measurement of oxygen uptake, metabolic efficiency and oxygen kinetics at each laboratory. Finally, we characterised the epigenetic profiles of all participants to explore the interaction between genetics, epigenetics and environment on observed phenotypes. All physiological measurement devices were tested for robustness and validity during hypobaric chamber and field testing prior to XE2. The data collection phase of XE2 has now been completed and analysis of these data been commenced.

## Discussion

Hypoxaemia is commonplace amongst critically ill patients yet optimal management strategies remain uncertain. Mechanisms that lead to beneficial adaptation, as opposed to maladaptation, need to be identified in order to develop individualised treatment strategies. The current standard response to hypoxaemia in critically ill patients is to aim for at least normal arterial oxygenation (and therefore, frequently supra-normal values are achieved); however, this may not be beneficial and in certain circumstances, may be injurious [17]. Whilst some augmentation of systemic oxygen delivery forms an important component of acclimatisation to high altitude, it fails to explain the marked observed differences in performance between individuals in this environment [18]. Similarly, there is little evidence to support the maintenance of normoxaemia in critically ill patients as a beneficial therapy [19].

Identifying cellular and metabolic differences between lowland individuals and those adapted over centuries to the hypoxia of high altitude may identify beneficial mechanisms of adaptation for subsequent evaluation in the clinical setting. Sherpa physiology may reveal novel target pathways that are amenable to pharmacological manipulation in the critically ill, such as the nitrate-nitrite-nitric oxide axis. Attempting to mimic the most effective human hypoxia-tolerant phenotype could provide new directions in critical care medicine.

The model of studying healthy volunteers ascending to altitude for the purpose of gaining insight into hypoxaemic patients is not without limitations. It is possible that mechanisms elicited at altitude may differ from those seen in pathology, environment factors other than hypoxia may confound data obtained in extreme conditions and differing levels of fitness may influence responses. However, comprehensive phenotype-genotype analysis in large groups of healthy volunteers exposed to an identical, graded hypoxic stimulus may unveil otherwise concealed mechanisms of importance [2,20].

In keeping with our core mission, the aim of XE2 is to create a large-scale biobank of samples linked to a phenotype database. This will be used to identify adaptive mechanisms and drive a translational research agenda. The consistency of data collection across the research expeditions run by the Caudwell Xtreme Everest Hypoxia Research Consortium has resulted in a unique bioresource for the study of human adaptation to hypoxia. Biomarkers and metabolic pathways can be exploited for patient benefit in an ongoing manner, and the resource can be interrogated repeatedly as new techniques evolve.

## Conclusions

The unique physiology of the Sherpa people may hold the key that unlocks the secret of successful hypoxic adaptation. The bioresource developed from XE2 (and CXE) provides a unique opportunity to explore human hypoxic adaptation and thereby drive improvements in the management of hypoxaemic critically ill patients in the years to come.

## Abbreviations

CASE: Centre for Altitude Space and Extreme Environment; CXE: Caudwell Xtreme Everest; CASE: Centre for Altitude Space and Extreme Environment; XE2: Xtreme Everest 2.

## Competing interests

The authors declare that they have no competing interests.

## Authors contributions

All authors (DM, EGK, DL, KM, RK, MM, MG) conceived the study, participated in its design and coordination, and helped draft the manuscript. All authors read and approved the final manuscript.

## References

[B1] West JB High life: a history of high-altitude physiology and medicine 1998 New York: Oxford University Press

[B2] Grocott M Montgomery H Vercueil A High-altitude physiology and pathophysiology: implications and relevance for intensive care medicine Crit Care 2007 11 203 10.1186/cc5142 17291330 PMC2151873

[B3] Grocott MP Martin DS Wilson MH Mitchell K Dhillon S Mythen MG Montgomery HE Levett DZ Caudwell xtreme Everest expedition High Alt Med Biol 2010 11 133 137 10.1089/ham.2009.1093 20586597

[B4] Grocott M Richardson A Montgomery H Mythen M Caudwell Xtreme Everest: a field study of human adaptation to hypoxia Crit Care 2007 11 151 10.1186/cc5921 17672886 PMC2206524

[B5] Levett DZ Martin DS Wilson MH Mitchell K Dhillon S Rigat F Montgomery HE Mythen MM Grocott MP Research Group CX Design and conduct of Caudwell Xtreme Everest: an observational cohort study of variation in human adaptation to progressive environmental hypoxia BMC Med Res Methodol 2010 10 98 10.1186/1471-2288-10-98 20964858 PMC2988011

[B6] Martin DS Ince C Goedhart P Levett DZ Grocott MP Abnormal blood flow in the sublingual microcirculation at high altitude Eur J Appl Physiol 2009 106 473 478 10.1007/s00421-009-1023-8 19333616 PMC2688617

[B7] Martin DS Goedhart P Vercueil A Ince C Levett DZ Grocott MP Changes in sublingual microcirculatory flow index and vessel density on ascent to altitude Exp Physiol 2010 95 880 891 10.1113/expphysiol.2009.051656 20418348

[B8] Levett DZ Radford EJ Menassa DA Graber EF Morash AJ Hoppeler H Clarke K Martin DS Ferguson-Smith AC Montgomery HE Grocott MP Murray AJ Acclimatization of skeletal muscle mitochondria to high-altitude hypoxia during an ascent of Everest FASEB J 2012 26 1431 1441 10.1096/fj.11-197772 22186874

[B9] Edwards LM Murray AJ Tyler DJ Kemp GJ Holloway CJ Robbins PA Neubauer S Levett D Montgomery HE Grocott MP Clarke K The effect of high-altitude on human skeletal muscle energetics: P-MRS results from the Caudwell Xtreme Everest expedition PLoS One 2010 5 e10681 10.1371/journal.pone.0010681 20502713 PMC2873292

[B10] Holloway CJ Montgomery HE Murray AJ Cochlin LE Codreanu I Hopwood N Johnson AW Rider OJ Levett DZ Tyler DJ Francis JM Neubauer S Grocott MP Clarke K Cardiac response to hypobaric hypoxia: persistent changes in cardiac mass, function, and energy metabolism after a trek to Mt. Everest Base Camp FASEB J 2011 25 792 796 10.1096/fj.10-172999 20978235

[B11] Levett DZ Fernandez BO Riley HL Martin DS Mitchell K Leckstrom CA Ince C Whipp BJ Mythen MG Montgomery HE Grocott MP Feelisch M The role of nitrogen oxides in human adaptation to hypoxia Sci Rep 2011 1 109 22355626 10.1038/srep00109 PMC3219423

[B12] Beall CM Reichsman AB Hemoglobin levels in a Himalayan high altitude population Am J Phys Anthropol 1984 63 301 306 10.1002/ajpa.1330630306 6731601

[B13] Beall CM Brittenham GM Strohl KP Blangero J Williams-Blangero S Goldstein MC Decker MJ Vargas E Villena M Soria R Alarcon AM Gonzales C Hemoglobin concentration of high-altitude Tibetans and Bolivian Aymara Am J Phys Anthropol 1998 106 385 400 10.1002/(SICI)1096-8644(199807)106:3<385::AID-AJPA10>3.0.CO;2-X 9696153

[B14] Marconi C Marzorati M Sciuto D Ferri A Cerretelli P Economy of locomotion in high-altitude Tibetan migrants exposed to normoxia J Physiol 2005 569 667 675 10.1113/jphysiol.2005.094979 16179365 PMC1464256

[B15] Erzurum SC Ghosh S Janocha AJ Xu W Bauer S Bryan NS Tejero J Hemann C Hille R Stuehr DJ Feelisch M Beall CM Higher blood flow and circulating NO products offset high-altitude hypoxia among Tibetans Proc Natl Acad Sci USA 2007 104 17593 17598 10.1073/pnas.0707462104 17971439 PMC2077056

[B16] Roach RC Bartsch P Hackett PH Oelz O Sutton JR, Houston CS, Coates G The Lake Louise AMS scoring consensus committee. The Lake Louise acute mountain sickness scoring system Hypoxia and molecular medicine 1993 Burlington, VT: Queen City Press 272 274

[B17] Martin DS Grocott MP Oxygen therapy in critical illness: precise control of arterial oxygenation and permissive hypoxemia Crit Care Med 2013 41 423 432 10.1097/CCM.0b013e31826a44f6 23263574

[B18] Martin D Windsor J From mountain to bedside: understanding the clinical relevance of human acclimatisation to high-altitude hypoxia Postgrad Med J 2008 84 622 627 10.1136/pgmj.2008.068296 19201935

[B19] Young JD Vincent JL Hypoxemia and mortality in the ICU Yearbook of intensive care and emergency medicine 2000 Berlin: Springer-Verlag 239 246

[B20] Grocott M Montgomery H Genetophysiology: using genetic strategies to explore hypoxic adaptation High Alt Med Biol 2008 9 123 129 10.1089/ham.2008.1012 18578643

